# Empirical Mode Decomposition-Based Deep Learning Model Development for Medical Imaging: Feasibility Study for Gastrointestinal Endoscopic Image Classification

**DOI:** 10.3390/jimaging12010004

**Published:** 2025-12-22

**Authors:** Mou Deb, Mrinal Kanti Dhar, Poonguzhali Elangovan, Keerthy Gopalakrishnan, Divyanshi Sood, Aaftab Sethi, Sabah Afroze, Sourav Bansal, Aastha Goudel, Charmy Parikh, Avneet Kaur, Swetha Rapolu, Gianeshwaree Alias Rachna Panjwani, Rabiah Aslam Ansari, Naghmeh Asadimanesh, Shiva Sankari Karuppiah, Scott A. Helgeson, Venkata S. Akshintala, Shivaram P. Arunachalam

**Affiliations:** 1 Bioinformatics and Computational Biology, University of Minnesota-Twin Cities, Minneapolis, MN 55455, USA; 2 Department of Radiology, Mayo Clinic, Rochester, MN 55905, USA; 3 Digital Engineering & Artificial Intelligence Laboratory (DEAL), Mayo Clinic, Jacksonville, FL 32224, USAaasthagoudel@gmail.com (A.G.);; 4 Department of Critical Care Medicine and Division of Pulmonary Medicine, Mayo Clinic, Jacksonville, FL 32224, USA; 5 Division of Gastroenterology & Hepatology, Department of Medicine, Johns Hopkins School of Medicine, Baltimore, MD 21287, USA

**Keywords:** empirical mode decomposition (EMD), gastrointestinal (GI) endoscopy, image classification, feature enhancement, image processing, deep learning

## Abstract

This study proposes a novel two-dimensional Empirical Mode Decomposition (2D EMD)-based deep learning framework to enhance model performance in multi-class image classification tasks and potential early detection of diseases in healthcare using medical imaging. To validate this approach, we apply it to gastrointestinal (GI) endoscopic image classification using the publicly available Kvasir dataset, which contains eight GI image classes with 1000 images each. The proposed 2D EMD-based design procedure decomposes images into a full set of intrinsic mode functions (IMFs) to enhance image features beneficial for AI model development. Integrating 2D EMD into a deep learning pipeline, we evaluate its impact on four popular models (ResNet152, VGG19bn, MobileNetV3L, and SwinTransformerV2S). The results demonstrate that subtracting IMFs from the original image consistently improves accuracy, F1-score, and AUC for all models. The study reveals a notable enhancement in model performance, with an approximately 9% increase in accuracy compared to counterparts without EMD integration for ResNet152. Similarly, there is an increase of around 18% for VGG19L, 3% for MobileNetV3L, and 8% for SwinTransformerV2. Additionally, explainable AI (XAI) techniques, such as Grad-CAM, illustrate that the model focuses on GI regions for predictions. This study highlights the efficacy of 2D EMD in enhancing deep learning model performance for GI image classification, with potential applications in other domains.

## 1. Introduction

The field of medical imaging, encompassing MRI, CT, ultrasound, endoscopy, and other modalities, has undergone a paradigm shift with the advent of deep learning (DL), which has enabled significant advances in image classification, detection, and segmentation across various medical domains, including oncology, cardiology, and neurology [1]. DL models, particularly convolutional neural networks (CNNs), have demonstrated the ability to learn detailed spatial patterns and subtle textural features directly from imaging data, enabling the development of automated diagnostic systems [2]. However, a critical challenge remains: these models often underperform when applied to early-stage disease detection, where image features are faint, nonlinear, and easily obscured by noise [3,4]. In such cases, poor feature extraction can lead to misclassification and missed diagnoses, especially when relying solely on raw image data.

One possible solution is to integrate signal-decomposition techniques as preprocessing within the DL pipelines. Methods such as wavelet transforms, empirical wavelet transforms, Fourier analysis, and empirical mode decomposition (EMD) decompose nonlinear high-dimensional signals into simpler sub-bands, boost the signal-to-noise ratio, and expose multiscale structures [5,6]. Among them, EMD is distinctive because it is adaptive and data-driven, requiring no preset basis functions. Although EMD is widely used in 1D signal decomposition, its application to 2D medical images has been largely overlooked. Despite their promise, decomposition-based DL frameworks remain underexplored in the medical imaging literature, particularly in classification tasks involving multi-class scenarios. Embedding a 2D EMD into the DL pipeline could lead to meaningful performance gains in challenging diagnostic tasks.

To investigate these prospects, we investigate the application of a 2D EMD-based preprocessing framework within the context of gastrointestinal (GI) endoscopic image classification. GI disorders, including ulcers, polyps, and esophagitis, are among the most prevalent global health issues, with GI cancers accounting for three of the eight most common cancers worldwide [7]. In the United States alone, GI-related illnesses cost over $135 billion annually and account for more than 54 million physician visits each year [8]. Capsule endoscopy (CE), a widely used diagnostic tool, captures over 55,000 frames per patient but poses interpretability challenges due to the volume and variability of the data [6,9]. Consequently, AI-driven systems can improve diagnostic precision, reduce human error, and enable early detection of GI diseases, ultimately leading to better patient outcomes and more effective treatment strategies [10].

**Review of state-of-the-art 2D EMD methods:**

A recent trend is observed in integrating different signal processing techniques into the deep learning-based pipeline. For instance, Mohapatra et al. [5] first applied the empirical wavelet transform (EWT) to decompose the image into several modes, and then these decomposed images were fed to a CNN model. They used a fraction of the HyperKvasir dataset to detect abnormal conditions of the GI tract. A similar approach was taken by Sethi et al. [6], where they combined wavelet and CNN to classify 8 classes from the Kvasir dataset. They reported improved performance for the Wavelet CNN over the conventional CNN. A major challenge to wavelet-based integration is that the choice of wavelet basis functions and decomposition levels can significantly impact the performance, requiring extensive experimentation and domain expertise. In addition, wavelet transforms may not effectively capture highly nonlinear heterogeneous patterns in the data, which can restrict the model’s ability to generalize well across different types of images.

Very few EMD-based approaches are available in the literature, especially for medical images. Some studies have attempted to extend 1D EMD to 2D. Among them, Yang et al. [11] proposed a 2D EMD framework for texture classification by detecting local extrema in grayscale images, building envelope surfaces with planar Delaunay triangulation, subtracting the mean envelope, and iteratively sifting until the stopping criterion is reached. In their follow-up study, Yang et al. [12] applied the same 2D EMD formulation to retinal fundus images for diabetic retinopathy analysis. Of the five extracted IMFs, they focused on IMF1 and computed nine multifractal features. Random Forest was used for feature selection, and k-means clustering was employed for unsupervised classification. Their evaluation was conducted on the DRIVE dataset (40 images) and a 60-image subset (20 healthy, 40 lesions) from a larger Kaggle dataset of 35,126 retinal images, achieving a reported accuracy of 96.24%. The authors acknowledged that the limited quantity and quality of the dataset images represent a major limitation of their method. Nguyen et al. [13] introduced a brain MRI enhancement method combining Bidimensional Empirical Mode Decomposition (BEMD) with anisotropic diffusion filtering and morphological operations for tumor detection and classification. Contrast was further enhanced through modified top-hat and bottom-hat transforms, and tumor regions were segmented using fuzzy C-means clustering. For classification, statistical features derived from wavelet packet coefficients were used with ensemble learning, achieving 76.7% classification accuracy. Senotova et al. [14] proposed a data augmentation method for pulmonary tuberculosis diagnosis in chest X-rays using Fast and Adaptive Bidimensional Empirical Mode Decomposition (FABEMD). Their approach decomposed X-ray images into bidimensional intrinsic mode functions (BIMFs) to remove low-frequency backgrounds and generate augmented training data. They evaluated the method on multiple public TB datasets, using an EfficientNetV2-M backbone. The results showed that FABEMD-based augmentation improved model performance across datasets, with the best effect observed at a background-removal threshold of 0.15. The authors noted that performance declined when the background-removal degree exceeded 0.20, indicating sensitivity to the chosen threshold and suggesting that the method is effective only within a limited operating range.

The above-mentioned methods for 2D EMD are generally limited to grayscale images and extract only a small number of IMFs. This presents a challenge for color-rich modalities such as endoscopy. Moreover, full decomposition of IMFs is required to capture the complete range of frequency components present in an image.

**Review of AI-based GI endoscopy image classification methods:**

Before deep learning came into the picture, earlier gastrointestinal endoscopic image classification applied techniques like neural networks [15], radial basis functions (RBF) [16], k-nearest neighbor (KNN) with support vector machine (SVM) [17], etc. Features are extracted using local binary patterns (LBP) [15], histogram of oriented gradients (HOG) [18], HSV color features [18], gray-level co-occurrence matrix (GLCM) [19], wavelet [20,21], etc. However, this paper focuses on deep learning-based approaches for gastrointestinal endoscopic image classification.

Cao et al. [22] proposed a six-layer CNN model to classify 5 types of lesions from wireless capsule endoscopy (WCE) images, achieving 95.15% accuracy. Each layer consists of one or two contiguous convolutions. While the authors reported high accuracy, metrics such as precision, recall, F1-score, and AUC were not provided, which could have offered a more comprehensive evaluation. Also, they augmented 1560 original images into 4260 training images and 900 test images. A more understandable result would have been obtained if they had reported evaluation results on the original test data. He et al. [23] prepared a dataset for the upper GI tract consisting of 3704 Esophagogastroduodenoscopy (EGD) images. They explored different deep learning models to classify 11 landmarks and reported that DenseNet-121 gave slightly better accuracy than Inception-v3, VGG-16-bn, VGG-11-bn, and ResNet-50. While the model differentiated most landmarks, the authors reported misclassification among anatomically similar regions (e.g., antrum and lower body) and reduced performance on low-quality or ambiguous images. Jha et al. [24] presented a thorough evaluation, comparison, and summary of the various methods introduced in the MediaEval Medico 2017, MediaEval Medico 2018, and BioMedia 2019 challenges. They emphasized that multi-class GI classification tasks face challenges in learning subtle medical features due to the lack of large datasets. This necessitates the improvisation of the classification frameworks. Lonseko et al. [25] applied a spatial attention mechanism to the CNN model to improve the classification of multi-site GI disease images. Their dataset consists of 12,147 multi-site, multi-disease GI images collected from public and private sources. They achieved an accuracy of 92.84%. The authors noted that further stability testing is needed to ensure consistent performance across diverse clinical settings. Wang et al. [26] used a pre-trained Inception-ResNet-v2 with a channel and spatial attention mechanism to classify esophagitis, polyps, and ulcerative colitis, trained and evaluated on the Kvasir dataset [7], achieving an accuracy of 92.5%. While the dataset includes 8 classes, their analysis focused on 3 classes. Additionally, 11% of the 3000 images were used for testing, with the rest allocated for training. Wei Wang et al. [27] proposed a two-stage endoscopic image classification method consisting of a lesion-aware CNN feature extraction module followed by a capsule classification network. They achieved an accuracy of 94.83% on the Kvasir dataset. However, they considered 6 out of the 8 classes and excluded two classes related to endoscopic polypectomy. Additionally, in their experiment, they excluded pixels of green patches that indicate where the endoscope is located. These patches pose a challenge for the classification task. Excluding green patch regions may have simplified the classification task, though this also limits insight into the model’s robustness under more realistic conditions. Consequently, the effect of those green patches on their deep learning model could not be evaluated. Ramamurthy et al. [28] proposed an ensemble network combining a pretrained EfficientNet-B0 and a custom CNN model called Effimix, which uses squeeze-and-excitation layers and self-normalizing activation layers. They achieved a 97% F1-score on HyperKvasir [29]. While the authors reported augmenting 10,662 images into a total of 23,000, using 15,264 for training and the remainder for testing, details regarding class-wise distributions or the proportion of original versus augmented images were not explicitly provided. Although augmentation can increase sample diversity, performance evaluation on augmented datasets may not always reflect real-world performance, particularly when original data distributions are not fully specified.

Therefore, the purpose of this work is to design and develop a new preprocessing technique based on 2D EMD that provides a full set of IMFs for deep learning applications, suitable for various prognostic, diagnostic, and treatment monitoring tasks in medical imaging, including color images. The utility of this technique is demonstrated using gastrointestinal endoscopic image classification, highlighting its potential to enhance the performance of various deep learning models.

## 2. Materials and Methods

### 2.1. Datasets

**Figure 1:**
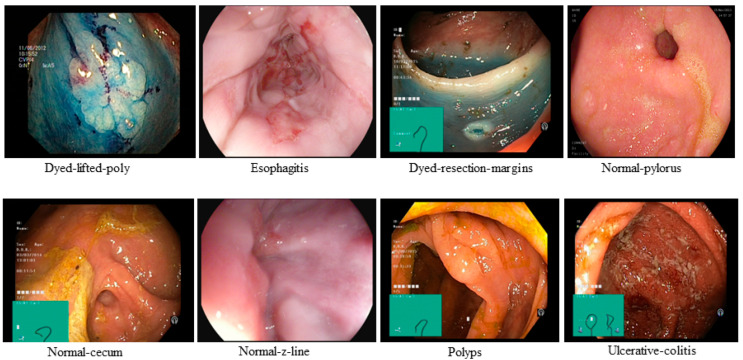
Samples of the Kvasir dataset.

This study utilized endoscopic images sourced from the publicly accessible Kvasir dataset [7], consisting of eight distinct image categories, each containing 1000 images. These categories include Polyps, Dyed-Resection-Margins, Normal-Pylorus, Normal-Cecum, Esophagitis, Dyed-Lifted-Polyps, Ulcerative-Colitis, and Normal-Z-Line. In our implementation, they are labeled as 0 to 7, respectively. The images were in different sizes and had significant black edges. Initially, all these images were manually cropped to remove the black borders, and subsequently, the resolution of the images was adjusted to 64 × 64. Out of 8000 images, 60% are used for training and validation, and 40% for testing. Therefore, 4800 images are allocated to training and validation, and 3200 images to testing. The 4800 images are further divided into 70% for training and 30% for validation. This results in 3360 images for training and 1440 images for validation. Figure 1 shows some samples of the Kvasir dataset.

### 2.2. One-Dimensional EMD Calculations

Empirical Mode Decomposition (EMD) stands out as a sophisticated method in signal processing, primarily employed for dissecting intricate nonlinear and non-stationary signals. Originating from the ingenuity of Norden E. Huang and his dedicated research team during the late 1990s [30], EMD emerged as a pivotal technique for dissecting signals into a finite collection of oscillatory constituents recognized as Intrinsic Mode Functions (IMFs). The utility of EMD transcends disciplinary boundaries, with its applications extending across diverse domains such as biomedical signal interpretation, financial market forecasting, and geophysical data analysis.

An IMF, denoted as *c_i_*(*t*), satisfies the following conditions:

- The number of extrema (maxima and minima) and zero crossings is either equal or differs by at most one throughout the signal.
- At any point, the mean value of the envelope defined by the local maxima *U_i_*(*t*) and the envelope defined by the local minima *L_i_*(*t*) is zero:

(1) $$Mean(U_{i}(t),\;L_{i}(t))=0$$

The decomposition process involves the following steps:

a. **Sifting Process:** Starting with the original signal *x*(*t*), a sifting process is applied iteratively to extract one IMF at a time. This involves identifying local extrema and interpolating between them to obtain upper *U_i_*(*t*) and lower *L_i_*(*t*) envelopes. The mean of these envelopes, *m_i_*(*t*), is then subtracted from the original signal to obtain the first IMF *c*_1_(*t*):

(2) $$c_{1}(t)=x(t)-m_{1}(t)$$

b. **Residue Calculation:** After obtaining the first IMF, the residue signal *r*_1_*(t)* is calculated as the difference between the original signal and the IMF:

(3) $$r_{1}(t)=x(t)-c_{1}(t)$$

c. **Iteration:** The process is repeated on the residue signal *r*_1_(*t*) until certain stopping criteria are met. At each iteration *i*, a new IMF *c_i_*(*t*) and corresponding residue *r*_i_(*t*) are obtained.

The decomposition process stops when one of the following conditions is met:

- The residue *r_i_*(*t*) becomes a monotonic function (i.e., it contains no more extrema).
- The number of extrema and zero crossings of the residue *r_i_*(*t*) becomes equal or differs by at most one.

The final decomposition consists of a set of IMFs *c_i_*(*t*) and a residue *r_n_*(*t*), where *n* is the total number of IMFs obtained. IMFs obtained through EMD are adaptive and data-driven, adapting to the local characteristics of the signal, and forming a complete orthogonal basis for a robust representation of the original signal. This method finds broad applicability across various domains where traditional signal processing techniques prove insufficient.

### 2.3. Two-Dimensional EMD-Based Design Procedure

EMD is conventionally used in a 1D vector context. However, our innovative contribution lies in the utilization of a 2D Empirical Mode Decomposition (EMD) framework. This unique approach involves decomposing images into their intrinsic mode functions, enhancing the controlled feeding process of IMFs, and removing oscillations at various scales from the original image. Our contribution is crucial for enhancing intrinsic features beneficial for AI model development. Moreover, the experiment is intelligently constrained to a maximum of three IMFs to strike a balance between complexity and execution time. In this study, we use a quasi-2D EMD formulation: the image is flattened for 1D EMD computation, and the resulting IMFs are reshaped back into 2D. After reshaping, however, the IMFs still reflect meaningful spatial patterns, which makes the decomposition suitable for our feature-enhancement pipeline. EMD calculation steps are shown below:

- **Flattening the RGB Image:** The original RGB image of size *H* × *W* × *3* is flattened into a 1D vector of size *H* × *W* × *3* × *1* using row-major ordering. This ensures that the pixel-wise spatial and channel information is preserved during flattening.
- **1D EMD Application:** Subsequently, a 1D Empirical Mode Decomposition (EMD) is applied to the 1D vector, resulting in the computation of 1D Intrinsic Mode Functions (IMFs).
- **Reshaping for Compatibility:** Each IMF is then reshaped back to the original image shape (*H* × *W* × *3*) to allow interpretation and visualization. It is important to note that higher-frequency IMFs may appear perceptually altered (e.g., inverted or noisy), which is a characteristic of the EMD.

**Figure 2:**
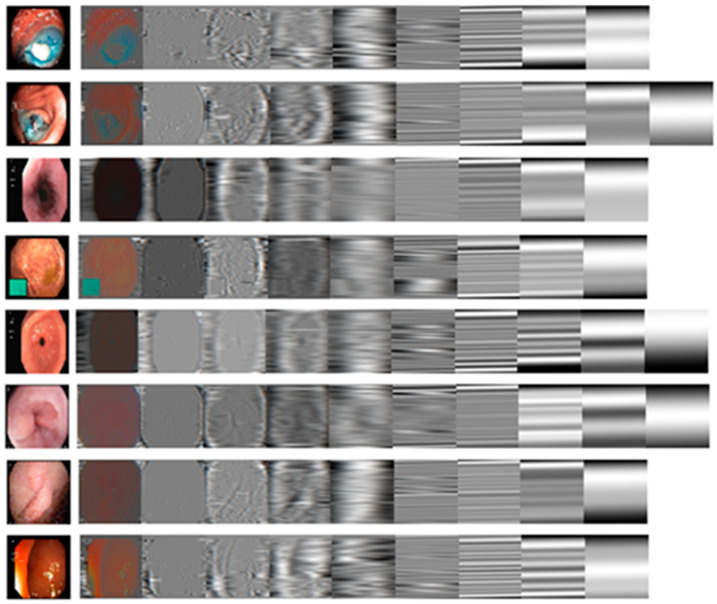
Representative examples of IMFs from a sample of each of the 8 GI endoscopic images using 2D EMD.

Figure 2 depicts instances of IMFs obtained from a selection of eight GI endoscopic images using 2D EMD.

In our design procedure, we generated enhanced images by subtracting the last few IMFs from the original image. This action offers several benefits:

- **Noise Reduction:** The last IMFs primarily capture low-frequency structures or trends in the image. Subtracting them from the original image removes slow-varying background information or illumination bias, thereby enhancing the contrast and local features relevant to deep learning.
- **Enhanced Feature Extraction***:* The removal of noise allows the AI model to focus more on the essential features of the image, leading to more accurate feature extraction and classification.
- **Simplification of Image:** Subtraction of the last few IMFs simplifies the image by removing unnecessary oscillations and variations, making it easier for the AI model to learn and generalize from the data.
- **Improved Contrast:** Removing noise and unnecessary details can improve the overall contrast of the image, making it easier for the AI model to distinguish between different regions and features.
- **Optimized Input for AI Models:** The subtraction of the last few IMFs can be seen as a preprocessing step that optimizes the input data for AI models, potentially leading to better performance and accuracy.

To showcase the effectiveness of 2D EMD in enhancing performance, we integrated our designed 2D EMD into a deep learning pipeline. We conducted experiments using four different deep learning models to evaluate the impact of 2D EMD. The selected models for evaluation were ResNet152 [31], VGG19bn [32], MobileNetV3L [33], and SwinTransformerV2S [34]. Here, ‘bn’, ‘L’, and ‘S’ mean batch normalization, large architecture, and small architecture. Models are taken from PyTorch’s website (https://pytorch.org/vision/stable/models.html, accessed on 5 December 2025). We did not use pretrained models, as our goal is to show how 2D EMD can be effectively used to increase the performance of deep learning models.

### 2.4. Training and Inference

All experiments were conducted on Google Colab Pro+ using PyTorch (version 2.9.0+cu126). Weight updates were performed using the Adam optimizer [35], with an initial learning rate set to 1 × 10^−3^ and weight decay to 1 × 10^−3^ to minimize losses. We employed the ReduceLROnPlateau technique, a learning rate scheduling method, to reduce the learning rate when the specified metric did not improve for a period longer than the specified patience value. We set the decreasing factor to 0.1 and the patience to 10. Using a batch size of 32, each model was trained for 50 epochs, with validation loss being monitored throughout. We continuously saved and updated the checkpoint whenever the validation loss decreased, ensuring that only the best checkpoint was used for evaluation during inference. Cross Entropy was chosen as our loss function, given the multi-class image classifications. For multi-class problems, the cross-entropy loss, *L*, is described as follows:

(4) $$L=-\sum_{i=0}^{n}\left(y_{i}\times log(p_{i})\right)$$

Here *y_i_* is the actual label and (*p_i_*) is the predicted probability for each class *i.*

## 3. Performance Metrics

In our research, we utilized multiple assessment metrics, including accuracy, precision, recall, and the F1-score, to assess the performance of the classifiers. The mathematical expressions for these evaluation metrics are depicted in Equation (1) through Equation (4). In these equations, *TP*, *TN*, *FP*, and *FN* represent True Positive, True Negative, False Positive, and False Negative, respectively. Here are the details pertaining to each definition:

(5) $$Accuracy=\frac{TP+TN}{TP+TN+FP+FN}$$

(6) $$Precision=\frac{TP}{TP+FP}$$

(7) $$Recall=\frac{TP}{TP+FN}$$

(8) $$F1-Score=2\times \frac{Recall\times Precision}{Recall+Precision}$$

We also used Receiver Operating Characteristic (ROC) and Area Under the Curve (AUC) for evaluation. A brief description of them is provided below.

**Receiver Operating Characteristic (ROC):** The Receiver Operating Characteristic (ROC) curve is a pivotal tool in assessing classification model performance, particularly in binary classification tasks. It illustrates the balance between sensitivity and specificity across various classification thresholds, offering a graphical representation of a model’s ability to distinguish between positive and negative instances. An ideal ROC curve hugs the upper-left corner, reflecting high sensitivity and low false positive rates, while a curve close to the diagonal line signifies performance akin to random guessing. By providing valuable insights into a model’s discriminatory power, the ROC curve enables researchers and practitioners to evaluate and compare different models’ performance across diverse classification thresholds.

**Area Under the Curve (AUC):** The Area Under the Curve (AUC) is a pivotal metric that encapsulates the classification model’s efficacy in distinguishing between each specific class and all other classes collectively. This metric is calculated by integrating the Receiver Operating Characteristic (ROC) Curve, offering a comprehensive assessment of the model’s discriminatory power across various classes. A higher AUC value indicates superior performance in correctly classifying instances across different classes, while a lower value suggests potential limitations in the model’s ability to make accurate distinctions. Thus, the AUC serves as a valuable indicator of the model’s overall discriminatory ability.

## 4. Results and Discussion

We defined four configurations to analyze the results, denoted as NO, IM–IMF-1, IM–IMF-1-2, and IM–IMF-1-2-3 in Table 1. The first signifies that EMD was not applied, the second indicates that the last IMF was subtracted from the original image, the third implies that the last two IMFs were subtracted, and the fourth suggests that the last three IMFs were subtracted. For convenience, we will refer to them as cfg-1, cfg-2, cfg-3, and cfg-4. For the Swin transfer, we only considered the first two configurations due to the lengthy training time required. As depicted in Table 1, subtracting IMFs from the original image consistently produced superior results in terms of accuracy, F1-score, and AUC for all four deep learning models. The best outcome was attained by ResNet152 and cfg-3, achieving an accuracy of 84.81%, an F1-score of 84.70%, and an AUC of 91%. The top performance without EMD was achieved by MobileNetV3L, yielding an accuracy of 79.63%, F1-score of 78.84%, and AUC of 88%. All EMD-based approaches achieved an AUC of 90% or higher. The last IMFs tend to encode low-frequency structures, background illumination drift, and slow-varying intensity trends. When these components are subtracted from the original image, the resulting representation suppresses global biases while enhancing local texture and contrast. This produces an image that more prominently exposes the diagnostically relevant patterns needed for deep learning, leading to performance gains. In addition, we performed similar experiments with three additional deep learning models–EfficientNet-B0 [36], InceptionResNetV2 [37], and SE-ResNet50 [38]. The results, presented in Appendix C, show that subtracting IMFs improves performance.

**Figure 3:**
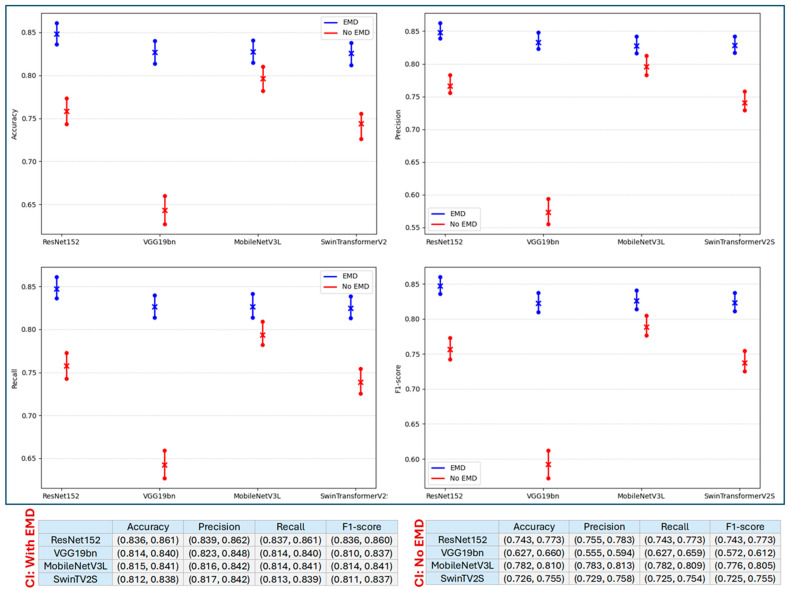
Confidence intervals (CIs) for accuracy, precision, recall, and F1-score across four deep learning models under two conditions: with EMD (blue) and without EMD (red). Vertical lines indicate the CIs, with circles marking the bounds and an “×” denoting the point estimate. Below the plots, two tables report the corresponding lower and upper 95% CIs (in parentheses) for all four metrics, separately for the with-EMD (**left**) and without-EMD (**right**) conditions.

Figure 3 shows the 95% confidence intervals (CIs) for four performance metrics across four deep learning models, evaluated under two conditions: with EMD (blue) and without EMD (red). Vertical lines indicate the CIs, with circles marking the lower and upper bounds and an “x” denoting the point estimate. Across all models and metrics, the blue intervals (with EMD) consistently lie above the red intervals (without EMD), indicating that incorporating EMD improves model performance and yields narrower, higher confidence intervals. In other words, EMD contributes not only to higher point estimates but also to greater stability across resamples.

**Figure 4:**
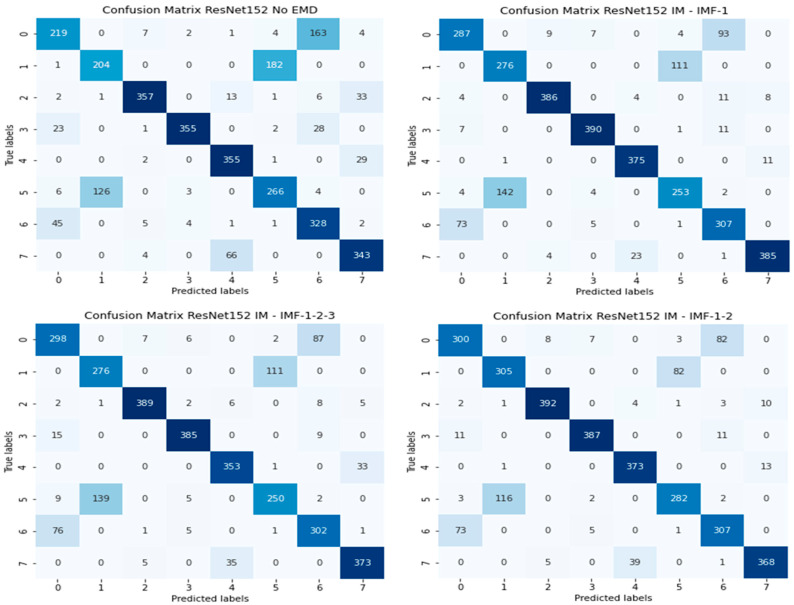
Confusion matrices for all four configurations and ResNet152.

**Figure A1:**
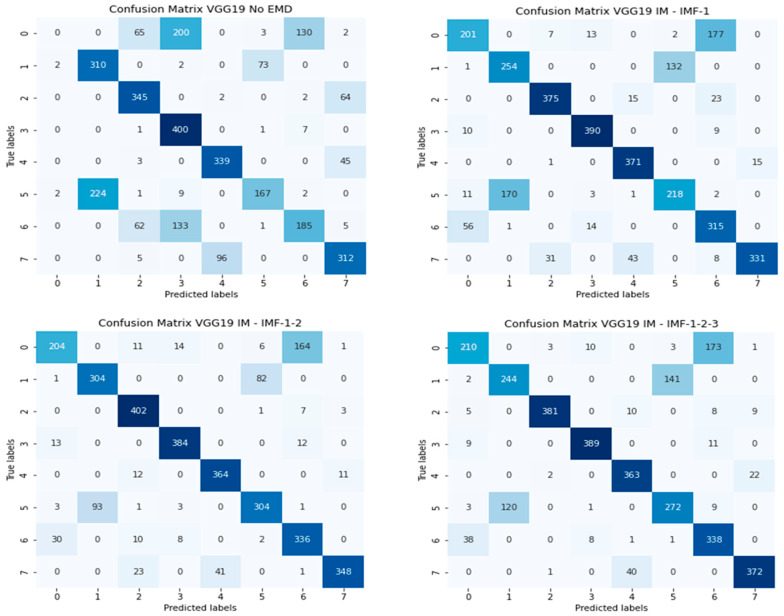
Confusion matrices for all four configurations and VGG19.

**Figure A2:**
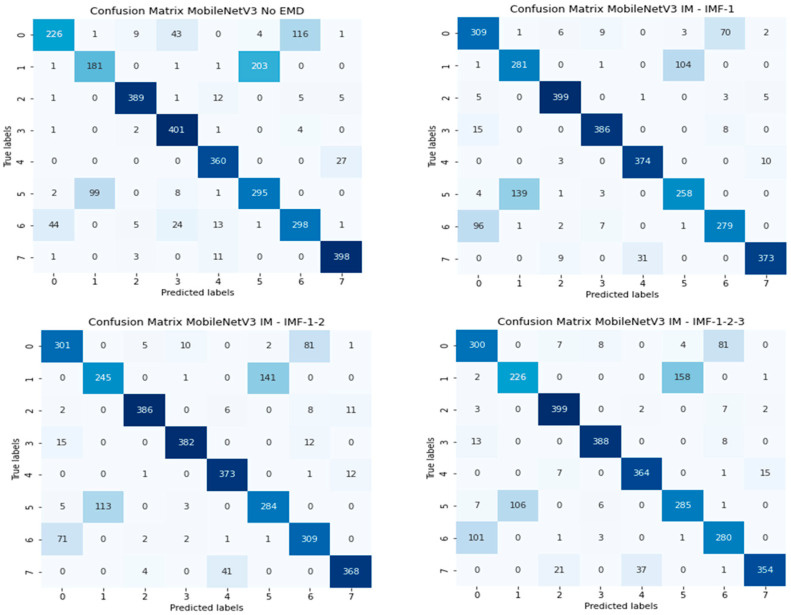
Confusion matrices for all four configurations and MobileNetV3L.

**Figure A3:**
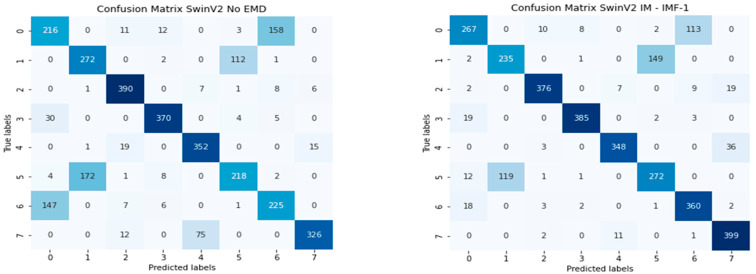
Confusion matrices for all four configurations and SwinTransformerV2S.

Figure 4 illustrates confusion matrices for all four configurations and ResNet152. Confusion matrices for the other deep learning models are presented in Appendix A (Figure A1, Figure A2 and Figure A3). It is evident that, for most classes, EMD-based approaches yielded higher true positives than those without EMD. Additionally, in the confusion matrix for VGG19bn provided in Appendix A, all polyps were misclassified without EMD.

**Figure 5:**
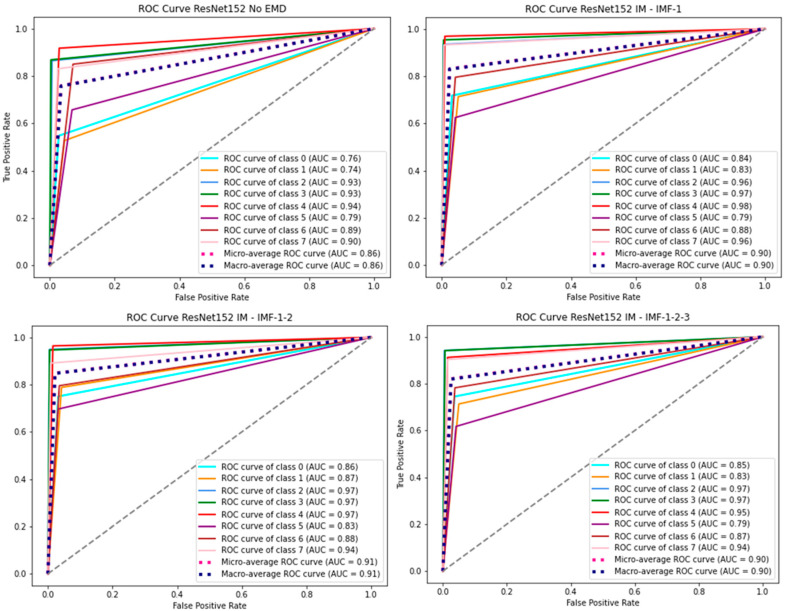
ROC curves for all configurations and ResNet152.

**Figure A4:**
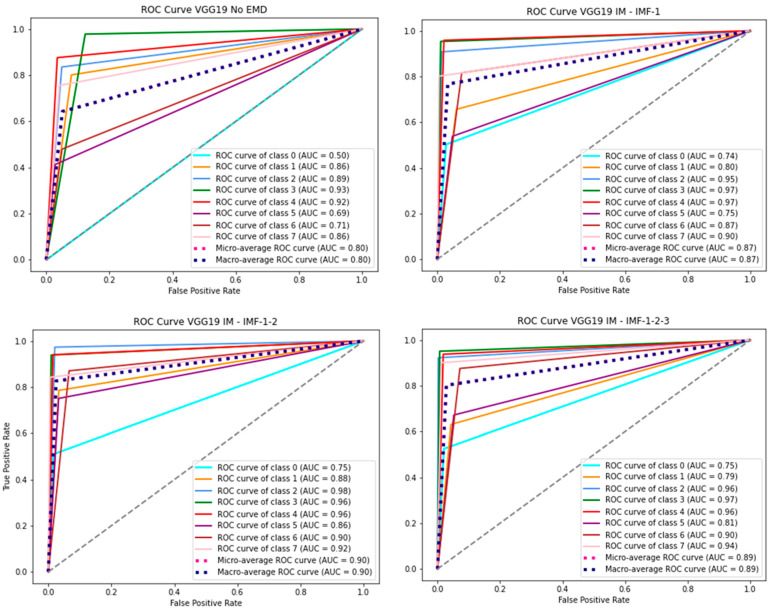
ROC curves for all configurations and VGG19bn.

**Figure A5:**
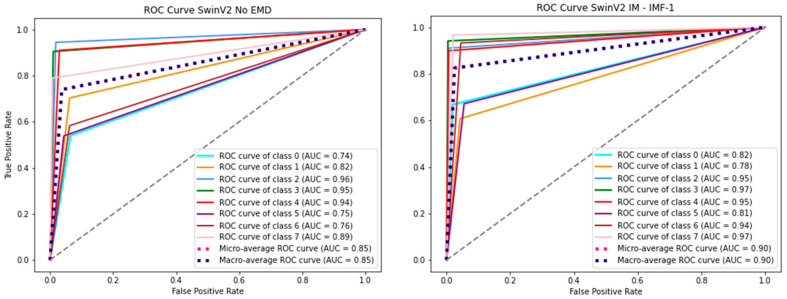
ROC curves for all configurations and SwinTransformerV2S.

**Figure A6:**
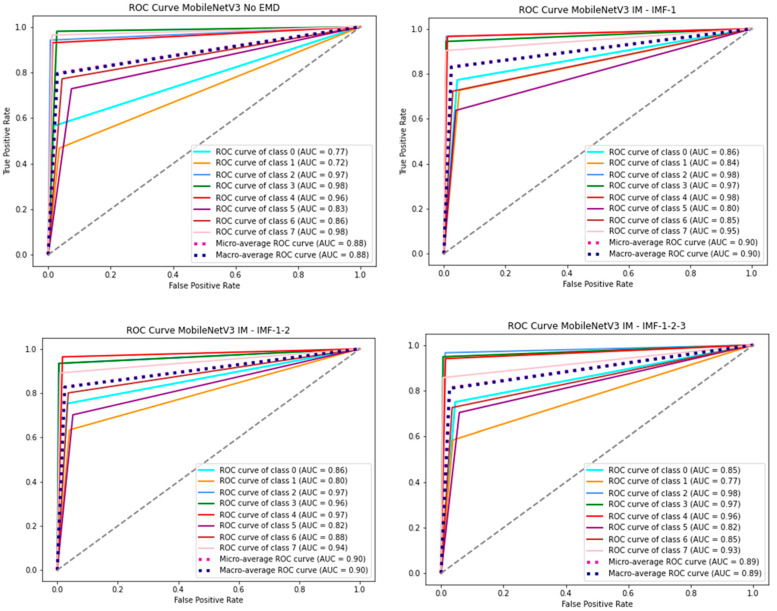
ROC curves for all configurations and MobileNetV3L.

Figure 5 illustrates the ROC curves for all four configurations and ResNet152. ROC curves for the other deep learning models are presented in Appendix B (Figure A4, Figure A5 and Figure A6). The ROC curves in this study were generated using one-hot encoded true and predicted class labels. As a result, the number of operating points on the ROC curve is limited, which may lead to atypical shapes, though they still provide valid class-wise AUC evaluations. The average AUC for cfg-2 with ResNet152 for all classes is 91%. Additionally, it is above 80% for all individual classes. However, without EMD, the average AUC is 86%, with three classes having AUC below 80%. As shown in Appendix B, the AUC for VGG19bn without EMD worsened further.

We then evaluated the results for individual classes. Table 2 presents the F1-scores for each class, with eight classes labeled from 0 to 7, as described in the Dataset section. Maximum values for each model are highlighted in bold. It is observed that, out of 112 F1-scores listed in the table, ‘without EMD’ performed marginally better in only 2 cases. In the remaining 110 cases, EMD-based approaches performed significantly better.

To highlight the performance improvement offered by EMD-based approaches, in Table 3, we tabulated the percentage changes in F1-score by integrating EMD compared to ‘without EMD’. A positive percentage change indicates an improvement compared to ‘without EMD’, while a negative sign indicates performance degradation due to EMD. For example, a 17.46% improvement indicates that ResNet152 improved the F1-score for class 0 (Polyps) by 17.46% by applying cfg-1 to the image before feeding it to the deep learning model. The maximum improvement observed with EMD is 63%. Out of 80 comparisons, there are only 8 instances of negative signs, indicating that ‘without EMD’ performed better. There were three comparisons where EMD-based and ‘without EMD’ produced the same result, and for the remaining 69 comparisons, EMD-based approaches showed better performance. To visualize the comparison, we plotted F1-scores for both with and without EMD for all four models.

**Figure 6:**
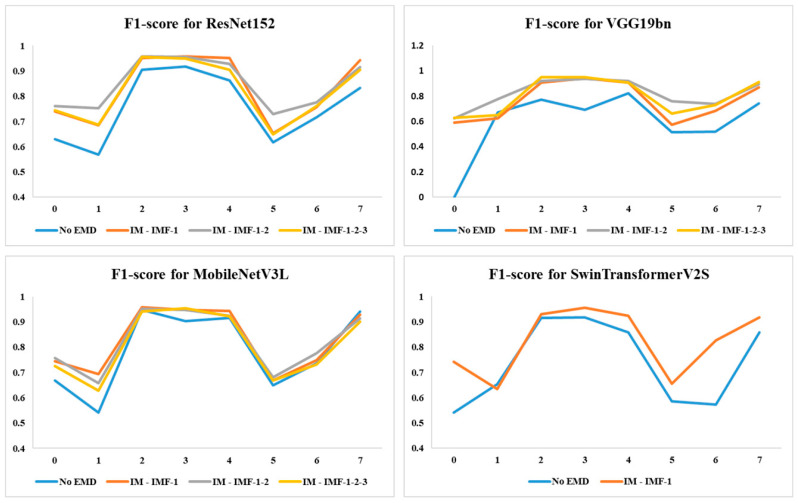
F1-scores for all four deep learning models.

In Figure 6, blue lines are used for ‘no EMD’. It is observed that in almost all cases, the blue lines remained below the other colored lines for all four models. This indicates that EMD-based approaches enhance the performance of deep learning models. We have demonstrated how EMD boosts the performance of four popular deep learning models and believe that it can enhance the performance of other deep learning models as well.

**Comparison with Existing 2D-EMD Approaches:** To contextualize our contributions, we compare our approach with several existing 2D EMD-based methods in terms of design, applicability, and integration with deep learning. The method proposed by Yang et al. [11,12] is limited to grayscale images and cannot be directly applied to RGB inputs without additional design considerations, which poses a challenge for color-rich modalities such as endoscopy. In addition, their pipeline incorporates extra computational stages, specifically, the Hilbert–Huang Transform and multifractal feature extraction, making the overall process more cumbersome and less suitable for integration into modern deep learning workflows. Their decomposition is fixed to five IMFs, potentially missing finer-scale information, and their evaluation is limited to small datasets (40 images from DRIVE and 60 from Kaggle), relying solely on machine learning techniques such as k-means clustering and random forest rather than end-to-end deep models. In contrast to our framework, Nguyen et al. [13] applied BEMD to grayscale brain MRI images, where the decomposition produced up to five intrinsic mode functions (IMFs), with the first four identified as noise-dominated. While this pipeline achieved high accuracy on the training set, the test accuracy dropped to around 76.7%, reflecting limited generalization. More importantly, because the approach was not designed for direct integration with deep learning, its reliance on grayscale inputs, manual feature engineering, and fixed IMF decomposition restricts scalability for sophisticated, multi-class image classification tasks. In comparison to the FABEMD-based augmentation approach introduced by Senotova et al. [14], our 2D EMD framework differs in both design and application. Their method focuses on background removal and augmentation of grayscale chest X-ray images, whereas our approach is designed to directly handle RGB endoscopic images, enabling broader applicability without conversion. FABEMD requires the computation of multiple BIMFs and relies on threshold-based selection of components to generate augmented data, which introduces additional sensitivity to parameter tuning and increases preprocessing complexity. In contrast, our 2D EMD pipeline integrates seamlessly with deep learning by selectively subtracting IMFs to enhance discriminative features. Moreover, while their experiments were limited to tuberculosis detection on a few public X-ray datasets, we demonstrate generalization in a multi-class gastrointestinal classification setting with large-scale benchmarks, achieving consistent improvements across four distinct deep learning architectures. Finally, FABEMD served primarily as a data augmentation strategy, whereas our method acts as a feature enhancement mechanism within the model pipeline, directly improving performance and interpretability through explainable AI techniques.

In summary, our proposed 2D EMD framework addresses key limitations of prior work by enabling full IMF decomposition on color images and integrating directly into deep learning pipelines. Through extensive experiments across diverse architectures and a large-scale dataset, we demonstrate consistent improvements in performance.

**Explainable AI (XAI):** The above discussion highlights that the EMD-based approach significantly enhances the performance of the deep learning model. This suggests that to make predictions, the model focuses on the gastrointestinal regions of the enhanced images generated by our 2D EMD. To verify this, we used explainable AI (XAI) to demonstrate where the model is focusing. Explainable AI is a field that aims to increase the transparency of AI models, particularly in understanding how they make decisions, especially when those decisions are critical.

This is achieved by developing methods that elucidate the rationale behind AI outputs. Given our model’s architecture as a convolutional neural network (CNN), we utilized Grad-CAM (Gradient-weighted Class Activation Mapping) [39] to visualize and interpret the model’s decisions. Grad-CAM helps in identifying which parts of the input data are crucial for the model’s predictions. This technique generates a heatmap highlighting the regions of the input image that have the most impact on the final prediction. It does this by calculating the gradient of the predicted class score with respect to the feature maps of the last convolutional layer. These gradients are then used to assign importance to each feature map, and the heatmap is created by combining the feature maps with a weighted sum, followed by a ReLU activation.

**Figure 7:**
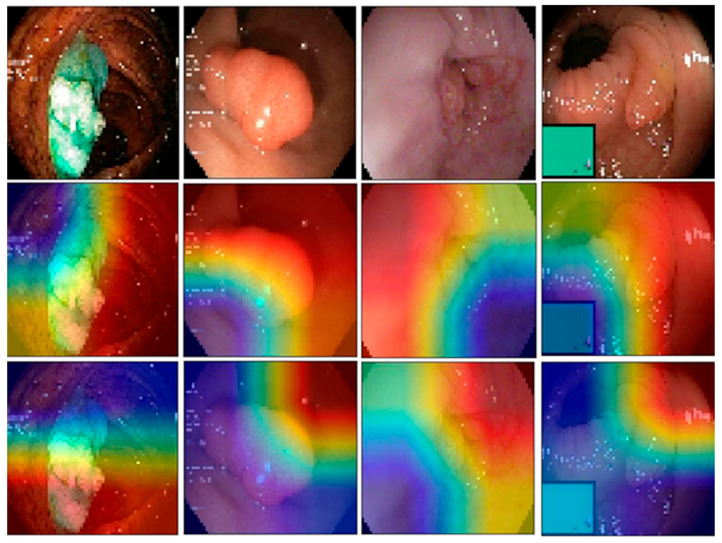
Comparison of Grad-CAM visualizations generated with and without EMD across representative gastrointestinal cases. Each column shows an original endoscopic frame (**top**), followed by the Grad-CAM heatmap from the baseline model (**middle**) and the EMD-enhanced model (**bottom**). The red zone indicates the more focused zone used for classification. Across dyed-lifted polyps, standard polyps, esophagitis, and additional mucosal lesions, the EMD-based visualizations exhibit more focused and anatomically consistent attention on clinically relevant regions, whereas the non-EMD heatmaps often produce broader activation that spills into normal mucosa.

In Figure 7, we present a side-by-side comparison of the original GI images and their corresponding Grad-CAM heatmaps generated with and without EMD. Each column displays the original image followed by the heatmaps from the baseline model (without EMD) and the EMD-enhanced model. In the first column, which presents a dyed and elevated polyp, the EMD-enhanced Grad-CAM shows a focused and well-bounded activation tightly aligned with the true lesion. In contrast, the corresponding non-EMD heatmap highlights the general region but spreads more broadly into adjacent mucosa. A similar trend is visible in the second column, where the EMD-based visualization concentrates cleanly on the polyp head, while the non-EMD version produces a more diffuse activation pattern that extends into normal tissue. In the third column, which presents an esophagitis case, the EMD-based heatmap is centered cleanly on the inflamed mucosa, capturing the erythematous region with good specificity. The non-EMD visualization also responds to the area but spreads more broadly into adjacent normal tissue, making the localization less precise. In the fourth column, the EMD-enhanced Grad-CAM again aligns closely with the true lesion by focusing its activation on the polyp-like protrusion and following its anatomical contour. The non-EMD version shifts its strongest activation upward and to the right, emphasizing mostly normal mucosa rather than the lesion and therefore drifting away from the clinically relevant region. Taken together, these examples illustrate that the EMD-enhanced model produces cleaner and more targeted activation patterns, particularly when the pathological region has a distinct and well-defined morphology.

**Figure 8:**
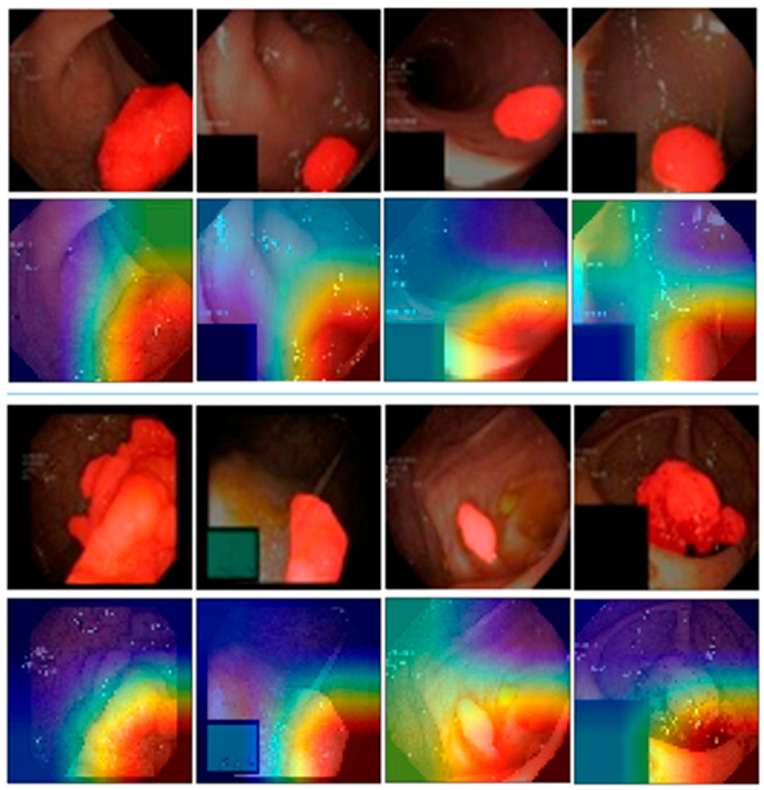
Additional XAI outputs. The 1st and 3rd rows show the original images with polyp masks overlaid in red. The 2nd and 4th rows display the corresponding heatmaps blended with the images. Red regions in the heatmaps indicate areas of greater model focus used for classification.

In addition, a set of polyp images with segmentation masks is collected from [33]. We overlay the masks in red on the polyp images, as shown in Figure 8 (1st and 3rd rows). The heatmaps in Figure 8 (2nd and 3rd rows) indicate that the model’s focus closely aligns with the polyp regions.

**Prospects and Future Directions:** Although the proposed framework demonstrates consistent improvements across four deep learning architectures, several opportunities remain for expanding and strengthening this line of research. First, while the Kvasir dataset offers balanced classes and is widely used in GI image analysis, it remains modest in size compared with large-scale endoscopy corpora. The current study serves as a proof of concept showing that 2D EMD can enhance feature richness even in limited-data settings. To further test generalizability, we are currently collecting a substantially larger endoscopic imaging dataset under an IRB-approved study. This expanded dataset will allow more rigorous evaluation across diverse patient populations, imaging devices, and real-world acquisition conditions.

A second direction concerns the adaptive behavior of intrinsic mode functions (IMFs). Because EMD is inherently data-driven, the number and characteristics of IMFs naturally vary with each image’s complexity. In this work, we explored subtracting the final few IMFs based on observed performance trends. Future extensions may integrate automatic IMF selection guided by frequency-domain signatures, entropy measures, or clinically meaningful heuristics. Such adaptive selection strategies could further improve discriminative power while reducing unnecessary computational cost.

Another important consideration is the relationship between model capacity and the benefits gained from EMD preprocessing. All comparisons were performed using the same architectures with and without EMD to ensure a fair assessment. While larger networks are generally capable of learning rich representations, our results suggest that EMD preprocessing reduces the burden on the model by presenting a cleaner, more structured input. This is particularly valuable for lightweight or deployable architectures, where EMD can serve as an efficient feature enhancer independent of network size.

Finally, because this mechanism is not task-specific, the denoising and feature enhancement benefits of EMD extend beyond classification. The same principle, which isolates meaningful structure while reducing oscillatory noise, can support many other imaging tasks, including lesion segmentation and diagnostic analysis across modalities such as ultrasound or pathology imaging. Moreover, spectral shifts across IMFs may hold potential for longitudinal tasks such as monitoring disease progression or assessing treatment response. Future investigations will explore these modality-agnostic applications and evaluate robustness across broader clinical domains.

Our study makes several significant contributions to the field of medical imaging for deep learning model enhancement using gastrointestinal endoscopy imaging examples:

- **Innovative Methodology:** We present a robust and computationally efficient strategy to implement full 2D Empirical Mode Decomposition (EMD) for image preprocessing. Unlike previous works that terminate decomposition prematurely or do not visualize the full set of IMFs, our method enables complete IMF extraction and integrates these into a deep learning pipeline with minimal computational cost, making it viable for real-world medical imaging tasks.
- **Improved Model Performance:** By subtracting the last few IMFs, we enhance image contrast and emphasize diagnostically relevant features. Our approach consistently improves accuracy, F1-score, and Area Under the Curve (AUC) across four popular deep learning models (ResNet152, VGG19bn, MobileNetV3L, and SwinTransformerV2). The integration of 2D EMD leads to notable enhancements in model performance, highlighting its effectiveness in GI image classification. For instance, an improvement in accuracy of approximately 9% for ResNet152, 18% for VGG19L, 3% for MobileNetV3L, and 8% for SwinTransformerV2 is observed.
- **Enhanced Model Interpretability:** Through explainable AI (XAI) techniques like Grad-CAM, we provide insights into the model’s decision-making process. The visualization of heatmaps demonstrates that the model focuses on GI regions for predictions, enhancing the interpretability of the AI model.
- **Reproducibility:** Our model uses publicly available data that allows other researchers to explore this methodology, ensuring the reproducibility of our results and facilitating further research and exploration in this area.
- **Potential for Broad Applications:** Although validated on GI endoscopic images, the proposed 2D EMD preprocessing technique is model-agnostic and modality-agnostic. The use of 2D EMD to enhance deep learning model performance could be applied to various image classification and early-stage disease detection challenges, contributing to advancements in AI-assisted diagnostics and image analysis using MRI, CT, ultrasound, endoscopy, and other imaging modalities.

## 5. Conclusions

Our study introduces a comprehensive and fully decomposed 2D EMD method for medical image preprocessing. By extensively evaluating and validating this novel technique within deep learning frameworks, we demonstrate significant improvements in model accuracy and diagnostic interpretability, marking an important advancement in AI-driven medical imaging diagnostics. The integration of 2D EMD consistently improves accuracy, F1-score, and AUC, with notable enhancements observed in ResNet152, VGG19bn, MobileNetV3L, and SwinTransformerV2S. Our approach not only enhances model performance but also provides insights into model interpretability through explainable AI (XAI) techniques like Grad-CAM, illustrating the model’s focus on GI regions for predictions. The experiments demonstrate that integrating EMD into the image-feeding process benefits endoscopy AI model development, and with a fast 2D EMD implementation, seamless integration into the digital endoscopy workflow is feasible at reduced computing power. This technique can be further expanded to all types of image classification and early-stage disease detection applications at different disease states. Although the dataset used in this study is moderate in size, the goal was to demonstrate the methodological advantage of integrating 2D EMD with deep learning. The 2D EMD framework enhances image representation by selectively removing low-frequency background components, thereby improving feature extraction and classification performance even with limited data. This preprocessing remains beneficial for larger datasets as well, serving as a flexible feature enhancement step that complements, rather than replaces, the learning capability of deep models. Overall, our study contributes to the advancement of AI-assisted diagnostics using the 2D EMD technique, offering a promising avenue for improving clinical workflows and patient care in the future.

## Figures and Tables

**Table 1:** Overall evaluation metrics for all classes. Bold values indicated the best values.

Model	EMD	Accuracy	Precision	Recall	F1	AUC
ResNet152	NO	75.84	76.65	75.77	75.63	86
	IM-IMF-1	83.09	83.05	83.00	82.95	90
	IM-IMF-1,-2	**84.81**	**84.79**	**84.75**	**84.70**	**91**
	IM-IMF-1,-2,-3	82.06	81.97	81.95	81.92	90
VGG19bn	NO	64.31	57.35	64.22	59.20	80
	IM-IMF-1	76.72	77.26	76.69	76.33	87
	IM-IMF-1,-2	**82.69**	**83.31**	**82.65**	**82.25**	**90**
	IM-IMF-1,-2,-3	80.28	80.90	80.19	79.92	89
MobileNetV3L	NO	79.63	79.62	79.37	78.84	88
	IM-IMF-1	**83.09**	**83.03**	**82.97**	**82.93**	**90**
	IM-IMF-1,-2	82.75	82.73	82.64	82.62	**90**
	IM-IMF-1,-2,-3	81.13	81.13	80.94	80.91	89
SwinTransformerV2S	NO	74.37	74.09	73.90	73.76	85
	IM-IMF-1	**82.56**	**82.81**	**82.45**	**82.31**	**90**

**Table 2:** F1-score for individual classes. Bold values indicate the best scores.

Model (F1)	EMD	0	1	2	3	4	5	6	7
ResNet152	NO	0.63	0.57	0.90	0.92	0.86	0.62	0.72	0.83
	IM-IMF-1	0.74	0.68	0.95	**0.96**	**0.95**	0.65	0.76	**0.94**
	IM-IMF-1,-2	**0.76**	**0.75**	**0.96**	**0.96**	0.93	**0.73**	**0.78**	0.92
	IM-IMF-1,-2,-3	0.75	0.69	0.95	0.95	0.90	0.65	0.76	0.90
VGG19bn	NO	0	0.67	0.77	0.69	0.82	0.51	0.52	0.74
	IM-IMF-1	0.59	0.63	0.91	0.94	0.91	0.58	0.68	0.87
	IM-IMF-1,-2	**0.63**	**0.78**	0.92	0.94	**0.92**	**0.76**	**0.74**	0.90
	IM-IMF-1,-2,-3	**0.63**	0.65	**0.95**	**0.95**	0.91	0.66	0.73	**0.91**
MobileNetV3L	NO	0.67	0.54	0.95	0.90	0.92	0.65	0.74	**0.94**
	IM-IMF-1	0.74	**0.69**	**0.96**	**0.95**	**0.94**	0.67	0.75	0.93
	IM-IMF-1,-2	**0.76**	0.66	0.95	**0.95**	0.92	**0.68**	**0.78**	0.91
	IM-IMF-1,-2,-3	0.73	0.63	0.94	**0.95**	0.92	0.67	0.73	0.90
SwinTrans.V2S	NO	0.54	**0.65**	0.91	0.92	0.86	0.59	0.57	0.86
	IM-IMF-1	**0.74**	0.63	**0.93**	**0.96**	**0.92**	**0.65**	**0.83**	**0.92**

**Table 3:** Change in F1-score by integrating EMD compared to not using EMD.

	EMD	0	1	2	3	4	5	6	7
ResNet152	IM-IMF-1	17.46	19.30	5.56	4.35	10.47	4.84	5.56	13.25
	IM-IMF-1,-2	20.63	31.58	6.67	4.35	8.14	17.74	8.33	10.84
	IM-IMF-1,-2,-3	19.05	21.05	5.56	3.26	4.65	4.84	5.56	8.43
VGG19bn	IM-IMF-1	59.0	−5.97	18.18	36.23	10.98	13.73	30.77	17.57
	IM-IMF-1,-2	63.0	16.42	19.48	36.23	12.20	49.02	42.31	21.62
	IM-IMF-1,-2,-3	63.0	−2.99	23.38	37.68	10.98	29.41	40.38	22.97
MobileNetV3L	IM-IMF-1	10.45	27.78	1.05	5.56	2.17	3.08	1.35	−1.06
	IM-IMF-1,-2	13.43	22.22	0.00	5.56	0.00	4.62	5.41	−3.19
	IM-IMF-1,-2,-3	8.96	16.67	−1.05	5.56	0.00	3.08	−1.35	−4.26
SwinTrans.V2S	IM-IMF-1	37.04	−3.08	2.20	4.35	6.98	10.17	45.61	6.98

## Data Availability

Publicly available data can be downloaded from https://datasets.simula.no/kvasir/ (accessed on 5 December 2025).

## Appendix C

Table A1 summarizes the performance of three additional deep learning models (EfficientNet-B0, InceptionResNetV2, and SE-ResNet50) under different preprocessing conditions with and without Empirical Mode Decomposition (EMD). EfficientNet-B0 and SE-ResNet50 were trained on images resized to 64 × 64, whereas InceptionResNetV2 required 96 × 96 inputs to avoid feature map dimensions shrinking below the kernel size in deeper layers. Across all models, subtracting Intrinsic Mode Functions (IMFs) consistently improved performance compared to using the original images alone.

**Table A1:** Overall evaluation metrics for additional deep learning models.

Model	EMD	Accuracy	Precision	Recall	F1	AUC
EfficientNet-B0	NO	91.47	91.98	91.34	91.39	95
	IM-IMF-1	93.28	93.37	93.20	93.23	96
	IM-IMF-1,-2	93.78	93.74	93.75	93.73	96
	IM-IMF-1,-2,-3	94.09	94.07	94.08	94.04	97
InceptionResNetV2	NO	92.91	92.89	92.88	92.85	96
	IM-IMF-1	93.66	93.62	93.62	93.60	96
	IM-IMF-1,-2	93.28	93.26	93.26	93.24	96
	IM-IMF-1,-2,-3	93.41	93.35	93.38	93.35	96
SE-ResNet50	NO	92.96	92.92	92.93	92.91	96
	IM-IMF-1	94.0	93.97	93.98	93.94	97
	IM-IMF-1,-2	93.34	93.38	93.33	93.28	96
	IM-IMF-1,-2,-3	94.16	94.15	94.14	94.09	97
